# Systemic and mucosal immune responses following oral adenoviral delivery of influenza vaccine to the human intestine by radio controlled capsule

**DOI:** 10.1038/srep37295

**Published:** 2016-11-24

**Authors:** Leesun Kim, C. Josefina Martinez, Katie A. Hodgson, George R. Trager, Jennifer R. Brandl, Erik P. Sandefer, Walter J. Doll, Dave Liebowitz, Sean N. Tucker

**Affiliations:** 1 Vaxart, Inc. 385 Oyster Point #9A, South San Francisco, CA 94080; 2 Scintipharma, 2201 Regency Rd # 403, Lexington, KY 40503.

## Abstract

There are several benefits of oral immunization including the ability to elicit mucosal immune responses that may protect against pathogens that invade through a mucosal surface. Our understanding of human immune biology is hampered by the difficulty in isolating mucosal cells from humans, and the fact that animal models may or may not completely mirror human intestinal immunobiology. In this human pharmacodynamic study, a novel adenovirus vector-based platform expressing influenza hemagglutinin was explored. We used radio-controlled capsules to deliver the vaccine to either the jejunum or the ileum. The resulting immune responses induced by immunization at each of the intestinal sites were investigated. Both intestinal sites were capable of inducing mucosal and systemic immune responses to influenza hemagglutinin, but ileum delivery induced higher numbers of antibody secreting cells of IgG and IgA isotypes, increased mucosal homing B cells, and higher number of vaccine responders. Overall, these data provided substantial insights into human mucosal inductive sites, and aided in the design and selection of indications that could be used with this oral vaccine platform.

Historically, mucosal vaccines are better at providing protection at a mucosal surface, eliciting either antibodies and/or T cells at the wet, open surfaces where the majority of pathogens invade. As an example, the oral polio vaccine generated more robust intestinal immunity than the injected inactivated vaccine^1^. Modern vaccines are being increasingly developed by recombinant DNA technology that allows expression of a key antigen. This is more efficient than the laborious trial and error methods of attenuating pathogens. While the mucosal system is partially linked, mucosal sites are highly compartmentalized and not all sites have equivalent potential to elicit immunity to vaccine antigens. Intestinal delivery is particularly challenging for a protein-based vaccine, given the proteolytic and tolerogenic nature of the intestinal space. All sites within the intestine do not have equal potential to generate effective immune responses to vaccines. There are differences in the mucosal layer^2^ (reviewed in ref. 3), availability of lymphoid clusters (e.g. Peyer’s patches)^3^,^4^,^5^, and commensal bacteria as one transits from the upper gastrointestinal tract to the colon^6^.

In order to deliver recombinant vaccines orally, we have created a platform technology that utilizes a replication-defective recombinant adenovirus type 5-vectored vaccine with a double stranded RNA adjuvant (rAd) that can be delivered in a tablet or capsule. Recombinant adenoviruses are well known for the ability to induce substantial antibody and T cell responses to the transgenic antigen. Unlike injected vector-based vaccines, studies in animals and humans have shown that oral vaccine delivery can circumvent pre-existing immune responses against adenovirus and generate substantial transgene specific immune responses^7^,^8^,^9^.

Because of the sensitivity of adenovirus to stomach acid degradation, enteric coatings are needed to protect the vaccine. Enteric coatings are designed to be impermeable at low pH and break apart at higher pH to allow the tablet contents to disperse. Specific enteric coatings are designed to open in specific regions of human intestine, but these cannot be modeled in animals due to differences in intestinal speed, pH, and immunobiology. Because of the potential differences in immunogenic activity between the lower and upper GI tract, it was not clear at first which tablet delivery site would be more effective. For this purpose, Radio Controlled Capsules (RCC), a controlled drug delivery method, allowing targeted delivery to a selected site in the human gastrointestinal tract, were employed^10^. The RCC technology has been used extensively in the pharmaceutical industry for oral drug formulation development, but has not to our knowledge, been used for vaccine studies. We used the RCC to determine the optimal delivery site for the vaccine.

We compared immune responses when the vaccine expressing influenza HA was targeted to release in the ileum versus the jejunum. Specifically, we investigated systemic HA-specific IgG response together with hemagglutination inhibition (HAI) and microneutralizing antibodies (MN) responses. In addition, we examined the HA-specific mucosaI IgA response and mucosal homing potential of antibody secreting cells (ASCs) in the peripheral blood in both the ileum and jejunum targeted groups. We showed that rAd oral vaccine targeted to either the ileum or the jejunum small intestinal sites was immunogenic in both cases. Importantly, the ileum-targeted release group generated more robust immune responses to HA than the jejunum by both serologic antibody responses as well as mucosal immune responses.

Results from this study were used to guide tablet development in subsequent clinical studies including the recently completed H1N1 enteric-coated tablet study^7^. In addition to the immunological benefits, an oral vaccine in a tablet form provides a more easily administered, more convenient and more broadly accepted alternative to an injectable vaccine.

## Methods

### Study design and participation/clinical protocol and enrollment

An open-label, Phase I, pharmacodynamic study was conducted between June 4 and October 10, 2013. Male volunteers age 18–49, in good health and able to swallow a large size triple 000 capsule were enrolled. The study was conducted in accordance with applicable Good Clinical Practice guidelines, the United States Code of Federal Regulations, and the International Conference on Harmonization guidelines. Following FDA clearance of the IND, IRB approval was obtained from Aspire IRB (Santee, California; AAHRPP accredited) before enrollment of subjects. Informed consent was obtained from participants. Vaccine administration took place at Scintipharma (Lexington, KY), where participants were fasted overnight. Capsules were given in the morning, and transit was monitored by gamma- Scintigraphy. Following release of the capsule contents, subjects were given a standardized meal. The clinical trial was registered with ClinicalTrials.gov on 2 January 2013. Clinical Trial registration number: NCT01761123.

### Vaccine

The vaccine was made under GMP conditions at Lonza, Houston as described before with the exception that the vaccine was not dried and put into tablets^7^. Rather, the material was dialyzed and concentrated over a sucrose buffer cushion by centrifugation. Each participant was given one dose of 1e11 infectious units (IU) in 0.9 ml of liquid, provided in a size 000 radio controlled capsule that was swallowed by each individual subject. The particle to IU ratio was 27.8 for this vaccine.

### PBMC isolation and cryopreservation

Blood was collected in K_2_ EDTA Vacutainer^**®**^ tubes (BD, Franklin Lakes, NJ) and PBMCs were isolated the same day using Lymphoprep™ tubes (Axis-Shield, Norway) in Scintipharma, Inc. PBMCs were frozen and thawed using serum free reagents according to the manufacturer’s instructions (Cellular Technology Ltd [CTL], Shaker Heights, OH).

### Antibody Secreting cells (ASCs)

Enzyme linked immunosorbent (ELISPOT) kits for IgG- and IgA-secreting B cells were performed according to manufacturer’s instructions (Mabtech, Mariemont, OH). Cells were cultured in triplicate wells in CTL-Test medium. HA protein (Protein Sciences Corp, Meriden, CT) was biotinylated and quantitated at Vaxart using a biotinylation kit and BCA kit (Pierce, Rockford, IL). Spots were counted at Zellnet Consulting Inc (Fort Lee, NJ).

### Antibody assays

Hemagglutination inhibition (HAI) and microneutralizing (MN) titers were performed by Focus Diagnostics (Cypress, CA) as described previously^11^. HAI and MN were measured against MDCK derived A/CA/07/2009 and egg derived A/CA/07/2009 respectively. HAI and MN titers less than 10 were marked as 5 as suggested by regulatory advice^12^.

### B cell immunophenotyping (Flow cytometry)

Immunophenotypical analysis was performed on cryopreserved PBMCs. All antibodies were obtained from BD Biosciences except anti-human IgA (rabbit polyclonal, DakoCytomation), AquaAmine (Invitrogen) and anti-human CCR9 (R&D Systems). The following antibodies were used: anti-CD19 (HIB19), anti-β7 (FIB504), anti-α4 (CD49d, 9F10), anti-CD27 (M-T 271), anti-IgG (G18–145), anti-CD38 (HB7), anti-β1 (CD29, MAR4) and anti-CD3 (SK7). Live and single cells were gated and CD3 + population was excluded. Data were collected on a Becton Dickinson (BD) LSR II cytometer using FACS-DIVA software (BD Biosciences) and analyzed using FlowJo software version Vx 0.6 (Tree Star Inc, San Carlos, CA).

### Nasal and fecal IgA ELISA

To collect nasal samples, 2.5 mL of saline was instilled into each nostril. The samples collected from each naris were pooled. Fecal samples were collected using stool collection tubes (Sarstedt). Both samples were immediately frozen at −70 °C. Each fecal sample was resuspended at 0.5 g/mL with PBS containing EDTA (Cellgro), PMSF (Cell Signaling Technologies) and soybean trypsin inhibitor (Sigma). Fecal extract was homogenized by vortexing samples. After centrifugation, the supernatants were collected and kept at −80 °C until the ELISA. In order to normalize for yield, total IgA was measured using a standard curve by ELISA (Mabtech). HA-specific IgA ELISA was performed as described^9^. Here, alkaline phosphatase conjugated human IgA antibody was used (Mabtech). HA-specific IgA ELISA assay was normalized to 10 μg of total IgA for all samples. Endpoint titers were chosen as the reciprocal of the highest dilution that had an absorbance value > 0.2 after the background (absorbance of the wells lacking a sample) subtraction. Higher than or equal to two-fold rise after vaccination was plotted.

### Statistical analyses

The Mann-Whitney non-parametric statistical test was used for comparing two unpaired groups, and the non-parametric Wilcoxon paired test was used for comparing pre and post immunized samples. Response rates were compared with the use of a two-tailed Fisher’s Exact test. *P* values of < 0.05 were considered significant. Analyses were performed using Prism v6.0 (GraphPad Software, La Jolla, CA).

## Results

### Study Population

**Figure 1:**
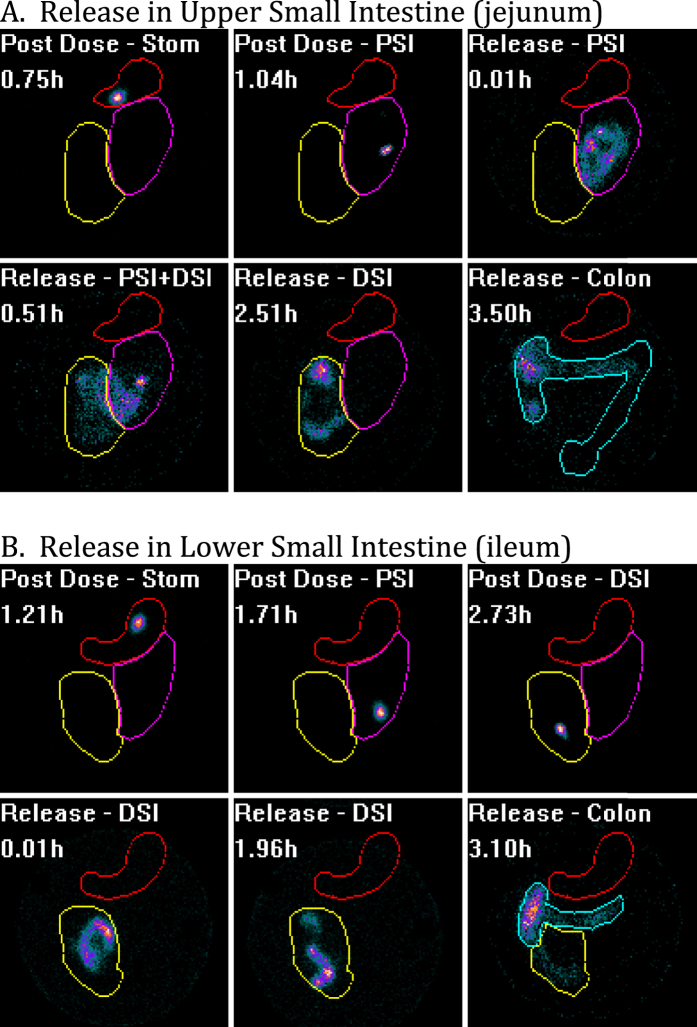
Scintigraphy visualization of subjects given vaccine released in the upper small intestine (**A**) versus the lower small intestine (**B**). Each subject swallows vaccine in a size 000 mechanical capsule loaded with liquid vaccine and a radiolabeled tracer. Post dose, one can visualize the capsule transiting through the stomach (Stom, red circle) and the intestine as a discrete spot because the radiolabeled tracer is all contained with the capsule. As the radiolabeled tracer proceeds through the proximal small intestine (PSI, purple circle) and the distal small intestine (DSI, yellow circle), the figures are labeled with such. Post release, the liquid contents are ejected from the capsule and dispersion of the material can be visualized as it spreads away from the capsule. When verified that the dispersed material has reached the colon (Colon, green outlined region), scintigraphy visualization is no longer required.

Twenty-four subjects were randomly assigned to receive the vaccine delivered to either the ileum or jejunum. Subjects were enrolled, fasted overnight, and given a liquid formulation of vaccine with a radioactive tracer in the mechanical capsule. The vaccine capsule’s progress through the digestive tract was monitored by gamma scintigraphy, and the capsule contents were released in the upper small intestine (jejunum) or lower small intestine (ileum) by radio signal that triggered the liquid contents to be ejected out of the capsule. Release was confirmed by scintigraphy; dispersion of the radioactive tracer demonstrated content release. Confirmed delivery to the ileum or jejunum occurred in 24 subjects out of 25 attempted. The subject with poor delivery had the contents of the RCC released in the colon or rectum, and was replaced such that 24 total subjects were monitored for immune responses. A representative figure showing the tracking of the capsule, followed by release in the ileum and jejunum are shown (Fig. 1).

### Antibody Secreting Cells

**Figure 2:**
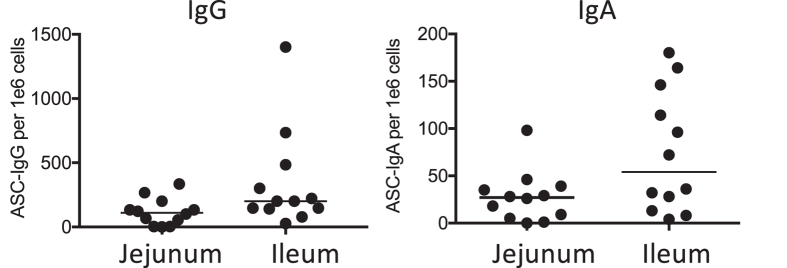
IgA and IgG ASC responses to HA following rAd oral vaccination to either the jejunum or ileum. Results are shown as numbers of HA-specific ASCs/10^6^ PBMC 7 days after vaccination. Each icon represents response level of one subject (**A,B**). Each bar represents median.

Influenza HA-specific IgG and IgA ASCs were measured in peripheral blood mononuclear cells (PBMCs) on days 0 and 7 after vaccination (Fig. 2). Background ASCs were not detectable on day 0. In the jejunum-targeted release group, a median of 27 IgA ASCs (95 CI: 5–39) and 111 IgG ASCs (95 CI: 3–200) each per 1 × 10^6^ PBMC were found at day 7 with one (IgG) and two (IgA) subjects out of 12 having no detectable ASC response. In the ileum-targeted release group, a median of 54 IgA ASCs (95 CI: 13–146) and 200 IgG ASCs (95 CI: 141–484) each per 1 × 10^6^ PBMC were found at day 7, with 12 out of 12 subjects responding for both IgA and IgG ASCs responses. The number of responders in both IgA and IgG ASCs in the jejunum-targeted group was less than in the ileum-targeted group, but not significantly. The HA-specific IgG ASCs numbers in the ileum-targeted group were significantly different from those in the jejunum-targeted group, but not significantly different in HA-specific IgA ASCs numbers (P = 0.023 (IgG) vs. P = 0.08 (IgA) by Mann-Whitney test).

### Serum Antibodies

Antibody responses (IgG) to HA were measured as described before^9^. Because of the significant prior exposure of humans to influenza, ELISA background titers are substantial, and the ability to see an increase in ELISA titers post immunization is challenging. In order measure changes in total IgG titers post immunization, we plotted OD versus dilution curves for each subject pre and post immunization and calculated an EC50 value for each curve. If there was an increase in the change of EC50 greater than 1.3, the subject was scored as a positive (1.3 was found in earlier studies to be associated with real gains versus background noise). Sample ELISA titer curves are included in the supplemental data. The RCC delivery of rAd vaccine to the intestine induced antibody responses to HA (by ELISA) in 12 out of 12 subjects in the ileum-released group and 9 out of 12 subjects in the jejunum-released group (data not shown). These results closely match the results for ASC response rate.

### Influenza Hemagglutination Inhibition (HAI)

**Figure 3:**
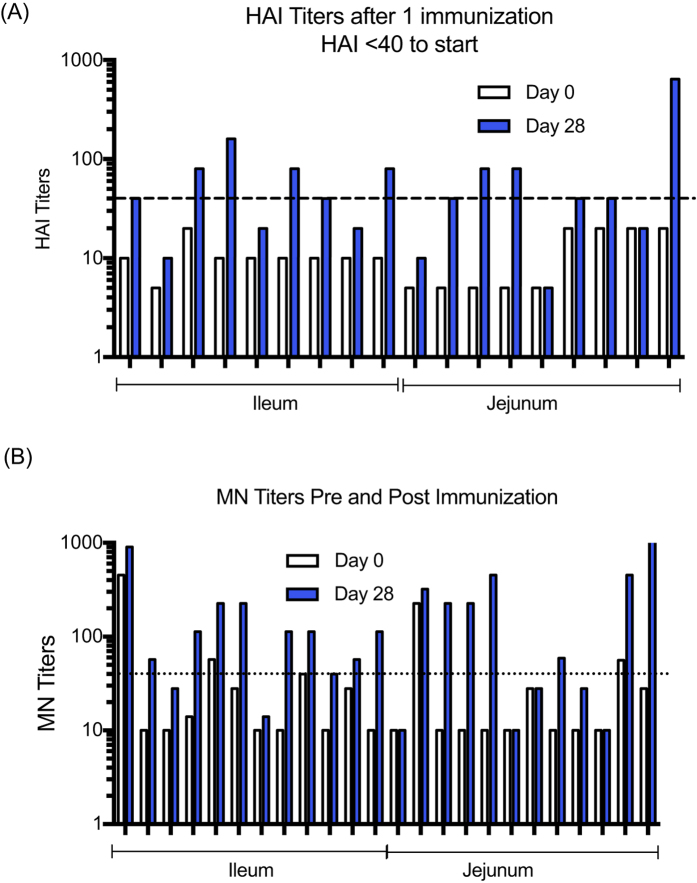
Antibody responses to HA following rAd oral vaccination to either the jejunum or ileum. (**A**) HAI titers to A/CA/07/2009 on days 0 and 28 after a single dose for the subjects that were not seroprotected (initial HAI < 40) at the start of the study. The line on the figure indicates a titer of 40 to show subjects seroprotected post immunization. (**B**) MN titers to A/CA/07/2009 for individual subjects on days 0 and 28 post immunization. The line shows the MN titer of 40.

HAI responses were measured on days 0 and 28 against MDCK derived A/CA/07/2009. The changes in HAI titers from baseline (D0 vs D28) following immunization can be seen for the subjects that were seronegative upon entering the study (Fig. 3). The vaccine elicited seroconversions (with an initial HAI < 40) in 67% of subjects given vaccine to the ileum and 44% of subjects given vaccine to the jejunum, but the difference in response rate was not significant.

### Microneutralizing (MN) Antibodies

Neutralizing antibody responses to influenza were measured by MN assay. Results suggest a trend where the ileum group appeared better than the jejunum group in terms of the frequency of responders (Fig. 3B). The frequency of subjects with 2 fold increases in MN titers in the ileum group (11 out of 12) was higher than the frequency in the jejunum group (7 out of 12), but not significantly different (P = 0.15 by Fisher’s exact test). After removing subjects that had MN titers greater than 40 prior to vaccination, as carried out by Faix *et al*.^13^, the geometric mean titers (GMT) and Geometric Fold Titer Response (GMFR) were calculated in the remaining subjects in a post hoc analysis (Table 1). These results show that serum neutralizing antibody titers to influenza were generated by oral immunization, with a greater than 5 fold increase in the GMT after immunization in the ileum group. While the frequency of responders in the jejunum group was lower than the ileum group, the GMT titers at Day 28 were greater than 80 due to four subjects having greater than a 20-fold increase in MN titers after immunization.

### Mucosal Immune Responses Measured by ELISA

**Figure 4:**
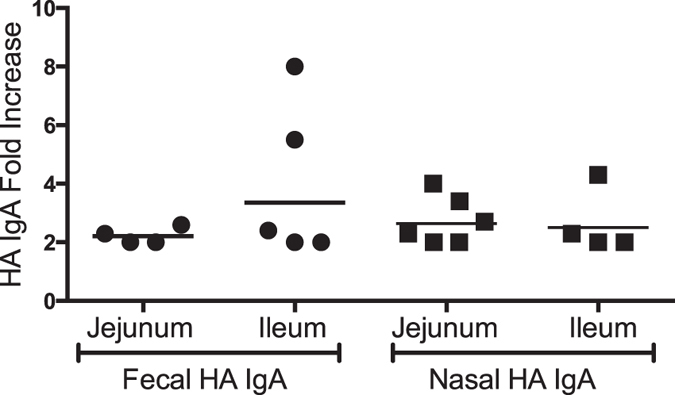
Fold increases in the anti-HA IgA responses to influenza HA following vaccine delivery by RCC to either the jejunum or ileum. Data are shown when a > 2-fold increase in GMT from day 0 to day 28 nasal and fecal samples had occurred.

Nasal wash and fecal samples were collected from human volunteers in this study. Total IgA was quantified by ELISA in order to normalize for yield. Following normalization, the fold rise in HA-specific IgA antibody titers was measured pre and post immunization. The background was high in both samples; slightly higher with nasal samples than fecal samples, but several subjects had a detectable specific IgA increase in titer post immunization. The number of subjects with detectable increases in anti-HA IgA titers (either in fecal or nasal IgA) was nine out of twelve in the ileum-targeted group and ten out of twelve in the jejunum-targeted group. Six subjects had increases in HA-specific IgA titers in nasal wash samples post-immunization in the jejunum group and four subjects had detectable IgA nasal responses in the ileum-targeted group (Fig. 4). The magnitudes were similar between groups. Four and five subjects had detectable increases in HA-specific IgA in fecal samples in the jejunum and ileum-targeted group, respectively, with a slightly higher response in the ileum delivery group (Fig. 4).

### Intestinal Mucosal Homing Receptors

Because of the issues with background signal and sample quality in the nasal and fecal IgA analysis, an alternative approach was taken to explore the mucosal homing potential of B cells in the peripheral blood post immunization. An ASC assay measures the numbers of HA specific pre-plasma B cells, but it does not determine tissue homing potential. Therefore, a flow cytometric approach was taken to analyze the capacity of ASCs (particularly IgA ASCs) to migrate to mucosal tissues post vaccination. The process of selective ASC homing is dependent on the binding of tissue-specific cell adhesion molecules. The most characterized adhesion molecule mediating an intestinal homing is the heterodimeric integrin α4β7^14^. Vascular endothelial cells that line the blood vessels within the intestinal mucosa constitutively express MAdCAM-1 (mucosal addressin cell adhesion molecule-1) and the migration of primed B cells into the intestinal mucosa is dependent on interactions between MAdCAM-1 and high levels of α4β7 integrin on B cells^14^,^15^,^16^,^17^.

**Figure 5:**
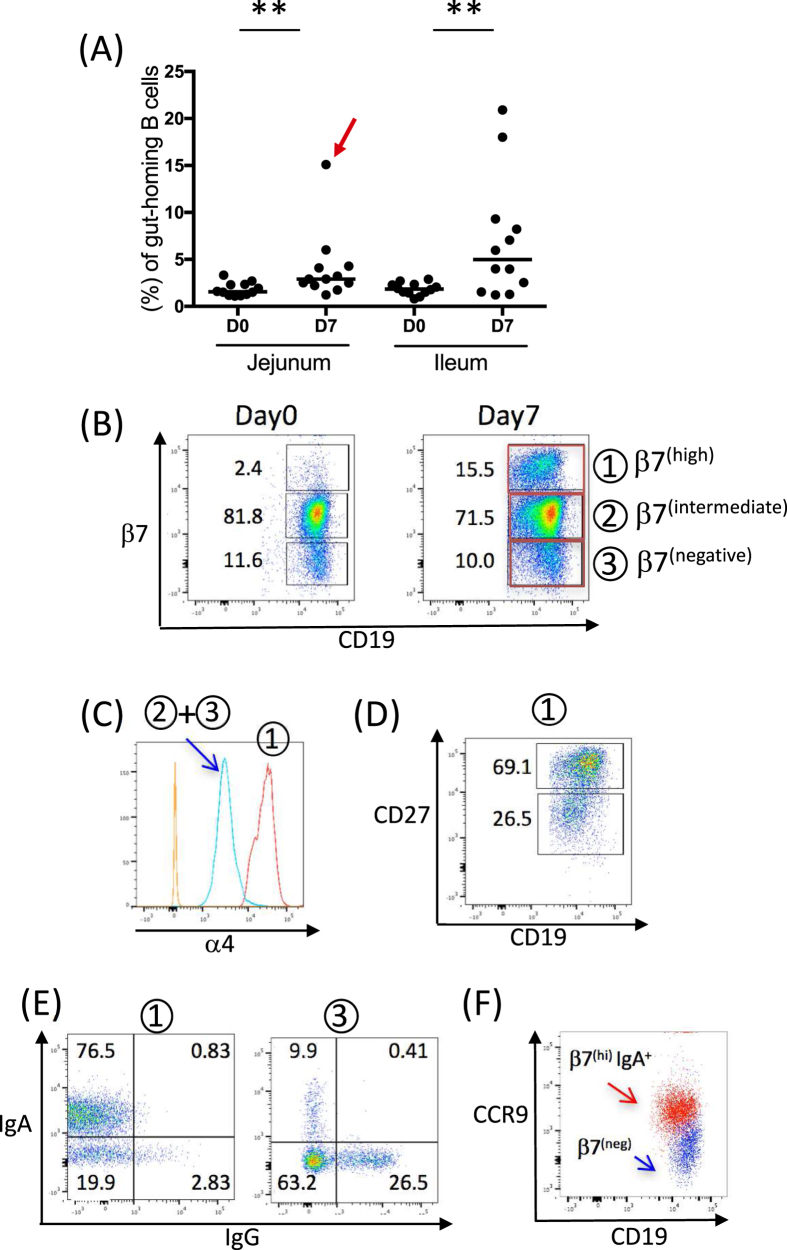
(**A**) The proportion of β7^(high)^ as a percentage of CD19^+^B cells in PBMCs are shown days 0 and 7 following vaccination in either the jejunum or ileum (**p = 0.0024 for the jejunum-targeted group and p = 0.0034 for the ileum-targeted group at day0 and day7, Wilcoxon test of paired t test). Each icon represents an individual subject. Bars represent the median of β7^(high)^ CD19^+^B cells. The red arrow indicates a subject that was analyzed for FACS data in Figs 5 and 6. (B) Samples of CD19 versus β7 staining are shown on day 0 and day 7 PBMCs. (**C**) Both β7^(intermediate)^ and β7^(negative)^ CD19^+^B cells expressed α4 integrin (blue line). The negative control (FMO) is shown in orange. CD19^+^β7^(high)^ cells have high expression of α4 integrin (red line). (**D**) On day7 following vaccination, β7^(high)^ B cells in PBMCs express CD27^(high)^ and CD27^(lintermediate)^. (**E**) Gating on β7^(high)^ and β7^(negative)^ B cells, surface IgA and IgG expression are shown. (**F**) β7^(high)^ IgA + B cells show CCR9 expression compared to β7^(negative)^ B cells. Population 1: day 7 β7^(high)^ CD19^+^B cells, population 2: day 7 β7^(intermediate)^ CD19^+^B cells, population 3: β7^(negative)^ CD19^+^B cells.

**Figure 6:**
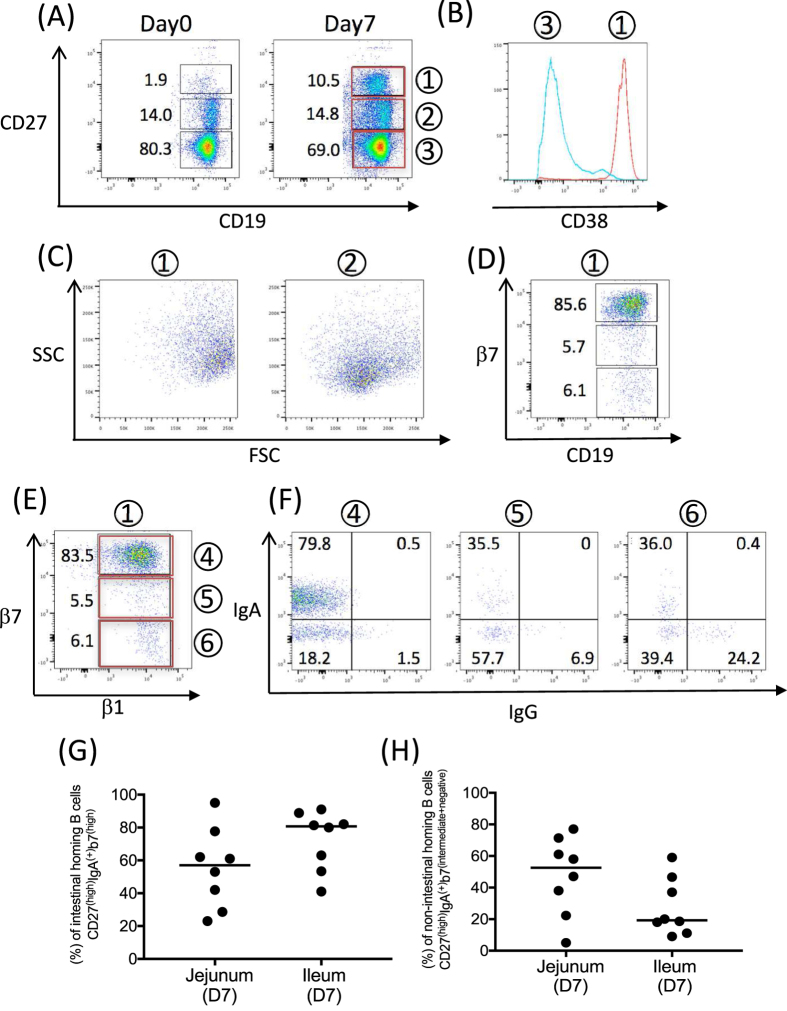
(**A**) After vaccination, CD27^(high)^ B cells appear in PBMCs. (**B**) CD27^(high)^ B cells express high levels of CD38 (red line) compared to CD27^(negative)^ (blue line). (**C**) The gated cells on day7 in (**A**) are shown for their FSC and SSC profile. CD27^(hi)^ cells are larger than CD27^(intermediate)^ B cells. (**D**) β7 expression on CD27^(high)^ B cells. The major sub population expressed β7^(high)^. (**E**) β1 integrin versus β7 integrin expression gated on CD27^(high)^ B cells. (**F**) IgA and IgG surface expressions gated on CD27^(high)^ B cells on day7. (**G**,**H**) comparison of intestinal and non-intestinal mucosal B cells in jejunum and ileum release groups.

We found a marked increase in β7^(high)^ CD19 + B cells (population 1) post vaccination in 21 out of 24 subjects (Fig. 5A). In the jejunum-targeted group, medians of 1.56% of β7^(high)^ B cells (95 CI: 1.2–2.4) on day 0 and 2.91% of β7^(high)^ B cells (95 CI: 2.2–4.3) on day 7 were observed. In the ileum-targeted group, medians of 1.84% of β7^(high)^ B cells (95 CI: 1.4–2.3) on day 0 and 4.98% of β7^(high)^ B cells (95 CI: 1.5–9.3) on day 7 were observed. Although the response was not significantly different between the two groups (two way ANOVA), a trend showing a higher induction of β7^(high)^ B cells in the ileum group was observed on day 7. Ten and eleven subjects (out of 12 each) showed increases in β7^(high)^ B cells after vaccination in the jejunum and ileum targeted groups, respectively. Flow cytometry is show with a representative subject, and used throughout the flow analysis in Figs 5 and 6, and marked in Fig. 5A with a red arrow. The representative subject is shown for β7 versus CD19 at days 0 and 7 to demonstrate the emergence of the β7^(high)^ population post immunization (Fig. 5B). β7^(intermediate and negative)^ B cells (population 2 and 3) expressed α4 integrin (Fig. 5C, blue line) and the corresponding β7^(high)^ B cells expressed high levels of α4 integrin (Fig. 5C, red line), suggesting rAd oral delivery generated α4^(high)^β7^(high)^ gut homing B cells.

In humans, circulating B cells can be broadly divided based on CD27 expression: naïve B cells do not express CD27, memory B cells express moderate levels of CD27, and pre-plasma cells express high levels of CD27^18^. After encountering its cognate antigen, a naïve B cell develops either into a memory B cell or a plasma cell precursor (pre-plasma cell). In the flow cytometric analysis, we found that newly appearing β7^(high)^ B cells contained both CD27^(high)^ pre-plasma cells and CD27^(intermediate)^ memory cells (Fig. 5D) and that β7^(high)^ B cells were highly enriched with surface IgA compared to the β7^(negative)^ B cell population (Fig. 5E). Tissue-specific chemokines work together with tissue-specific adhesion molecules to coordinate the migratory pattern of ASCs to target effector tissues. Epithelial cells in the small intestine secrete the chemokine CCL25 (also known as TECK, thymus-expressed chemokine), a ligand for CCR9^19^. β7^(high)^ IgA-bearing B cells expressed CCR9, suggesting that these B cells will likely home back to the small intestine (Fig. 5F).

### Non-Intestinal Mucosal Homing Receptors

Next, we examined the migratory capacity of pre-plasma B cells to the non-intestinal mucosal tissues. Most human blood pre-plasma cells express CD19^(+)^CD27^(high)^CD38^(high)^. A new population of CD27^(high)^ CD19^+^B cells (population 1) was generated following vaccination (Fig. 6A). They expressed high levels of CD38 (Fig. 6B). CD27^(high)^ B cells were larger and more granular in size due to the clonal expansion and antibody synthesis compared to the CD27^(intermediate)^ B cells (population 2), (Fig. 6C), which are likely small, resting memory cells. These data suggested rAd oral delivery generated CD19^(+)^ CD27^(high)^CD38^(high)^ pre-plasma B cells. A major subset of CD27^(high)^ B cells expressed high levels of β7, as expected following oral vaccination (Fig. 6D).

We examined β1 integrin and surface IgA and IgG expression from pre-plasma B cells (population1). In addition to the β7 integrin, β1 integrin is an alternate partner for the α4 integrin. We observed before that β7^(high)^ B cells co-expressed high levels α4 integrin and both β7^(intermediate)^ and β7^(negative)^ B cells expressed the α4 integrin. α4β1 binds to endothelial cells via the vascular cell adhesion molecule, VCAM-1^19^. Although the expression of VCAM-1 at various effector sites is not yet well documented, IgA bearing α4^+^β1^+^/α4^+^β7^(intermediate)^ and α4^+^β1^+^/α4^+^β7^(negative)^ B cells appear to have tropism to mucosal tissues outside the gut (that is mammary gland, salivary gland, respiratory tract and urogenital tract)^16^,^19^. All three subsets (β7^(high)^, β7^(intermediate)^ and β7^(negative)^, populations 4, 5 and 6 respectively) expressed the β1 integrin (Fig. 6E) and each subset had a substantial population of surface IgA or IgG, with the expression level of β7 correlating to increasing percentages of surface IgA and decreasing percentages of IgG (Fig. 6F).

Lastly, we compared (1) intestinal and (2) non-intestinal mucosal homing B cell populations between jejunum, and ileum release groups using the available samples remaining (12 of 12 jejunum release group, 10 of 12 ileum). We examined the percentage of β1^+^/β7^(high)^ and β1^+^/β7^(intermediate+negative)^ B cells from CD19^(+)^ CD27^(high)^ IgA^(+)^ B cells on day 7 in jejunum and ileum release groups (Fig. 6G,H). There were 2 subjects in ileum release group and 4 subjects in the jejunum release group did not show an increase of CD19^(+)^ CD27^(high)^ B cells on day 7 over day 0, but the number of responders in the jejunum group is not significantly different from ileum group by Fisher’s Exact test. Subjects with increases in the CD19^(+)^ CD27^(high)^ populations were plotted in Fig. 6G and H. The Ileum treated group trended higher for percentage of intestinal homing B cells (Fig. 6G), but the jejunum treated group trended higher in the generation of non-intestinal mucosal homing B cells (Fig. 6H). These were not significantly different (Mann-Whitney test).

**Figure 7:**
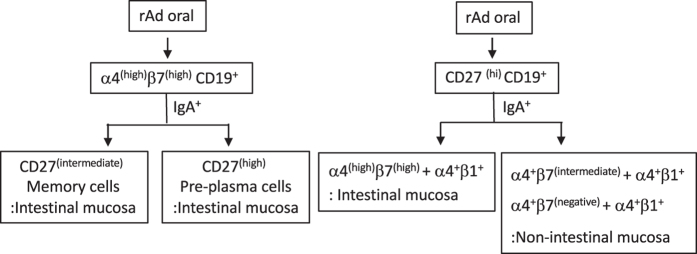
A schematic representation of the subsets generated after rAd oral vaccination. Population 1: day 7 CD27^(high)^CD19^+^B cells, population 2: day 7 CD27^(intermediate)^ CD19^+^B cells, population 3: CD27^(negative)^ CD19^+^B cells, population 4: CD27^(high)^CD19^+^β7^(high)^ β1^+^, population 5, CD27^(high)^CD19^+^β7^(intermediate)^ β1^+^, population 6: CD27^(high)^CD19^+^β7^(negative)^ β1^+^.

In summary, the oral vaccine generated a substantial B cell population with intestinal tropism that includes (1) α4^(high)^β7^(high)^CD27^(high)^IgA^(+)^ pre-plasma B cells and (2) α4^(high)^β7^(high)^CD27^(intermediate)^ IgA^(+)^ memory B cells. In addition, a smaller population of non-intestinal mucosal homing pre-plasma B cells (α4^+^β7^(intermediate)^/α4^+^β1^+^IgA^(+)^ and α4^+^β7^(negative)^/α4^+^β1^+^IgA^(+)^) were also generated (Fig. 7).

## Discussion

The use of RCC technology provided valuable information on the biological performance of the rAd oral vaccine released in liquid form, subsequently leading to the development of an effective enteric-coated tablet. Tablets are a desired format for drug and vaccine delivery due to easier administration, rapid distribution, and user preference. However, given the experimental design of the RCC study (direct delivery to the ileum or jejunum) and the willingness of the study participants, closer examination of human mucosal immune responses in humans was performed with this study.

The induction of antigen-specific B cell response after oral immunization is largely dependent on germinal centers in the gut-associated lymphoid tissues (GALT) consisting of Peyer’s patches (PPs) and isolated lymphoid follicles as well as in the gut-draining mesenteric lymphoid nodes (MLN). PPs are considered to be the major inductive sites and are more prevalent in the human ileum than the jejunum. This would suggest the ileum would be a more ideal region for vaccine release. In contrast, published studies in sheep demonstrated that jejunum delivery of rAd was more potent than ileum delivery for the ability to elicit immune responses against transgene^20^. This study using the RCC technology allowed us to answer questions about the potency of each site in humans.

We observed higher responder rates and higher HA-specific ASCs numbers when the rAd oral vaccine was released into the ileum. In terms of HAI titers, up to 67% seroconversion was seen with the ileum group. This rate is similar to that reported in our recently published paper using tablets with enteric coatings that target release to the ileum where 75% seroconversion was observed^7^. Given that injected commercial vaccines have obtained H1N1 seroconversions between 45 and 65% in subjects^21^, these results suggest that oral immunization with rAd could generate comparable response rates. The vaccine delivery to the ileum appeared to trend toward an improved antibody performance and higher α4^(high)^β7^(high)^ B cell induction compared to delivery to the jejunum. This finding suggests that more efficient immune induction might occur when the rAd oral vaccine was released in the ileum. There are several reasons why the ileum might be more efficient in immune induction, including the observation that the area has denser lymphoid clusters. The jejunum site was less efficient, but both IgG and IgA ASCs responses as well as α4^(high)^β7^(high)^ mucosal B cells were induced in individual subjects in this group. For the vaccine released in the jejunum, the induction sites could be local PPs in the jejunum, or induction sites could be PPs in the ileum if the rAd reached the ileum via jejunum release and transit. Another possible inductive site can be MLN. The antigen could have drained into the MLNs and B cells could be primed in MLN instead of GALT^16^.

After naive B cells are primed in GALT or MLNs, they leave the lymph nodes via draining lymphatics and re-enter the blood circulation via the thoracic duct. The α4^(high)^β7^(high)^ integrins are intestinal specific homing receptors that direct primed IgA positive B cells into the effector tissue, the intestinal lamina propria. This α4^(high)^β7^(high)^ phenotype is predominantly induced after oral immunization, whereas systemic immunization preferentially induced CD62L expression in humans^22^. Consistently, we found a large proportion of α4^(high)^β7^(high)^ B cells in peripheral blood after intestinal delivery of our vaccine (Fig. 5A and B). While no placebo subjects were enrolled in this study, the increase in this α4^(high)^β7^(high)^ phenotype was also observed in our vaccine tablet participants and not in the placebo treated subjects (L. Kim, unpublished results). CCL25, the ligand for CCR9 is constitutively expressed at high levels by epithelial cells of the small intestine^23^. We found CCR9 was co-expressed with cells of the α4^(high)^β7^(high)^ phenotype (Fig. 5F).

Intestinal dendritic cells (DCs) have the capacity to drive the expression of CCR9 and α4β7 on lymphocytes through retinoic acid (a metabolic product of vitamin A) production^24^. In addition, together with unique intestinal cytokine milieu, dendritic cells play an essential role for IgA induction in the intestines. Thus co-expression of α4β7 and CCR9 targets the efficient recruitment of IgA-expressing plasmablasts to the small intestinal lamina propria, and likely represents the eventual destination of the recently activated β7^(hi)^ cells in our study.

We were able to measure HA-specific IgA with fecal and nasal samples in several subjects post immunization. In terms of fecal analysis, we found HA-specific IgA increases in several subjects, but not all. One reason why human fecal sampling is a less reliable method of studying mucosal secretory IgA responses is because the human intestines contain various proteolytic enzymes^25^,^26^. We added a mixture of protease inhibitors to the fecal extraction buffer to help with recovery of fecal IgA, but in some cases the sample may have been held for periods over 24 hours before processing. For ileum vaccine delivered subjects, 92% had a detectable α4^(high)^β7^(high)^population in the peripheral blood post immunization, but only 42% of subjects had a detectable increase in HA specific IgA in fecal samples. In terms of nasal IgA, we also observed some subjects with an increase in specific IgA to HA post immunization. Although background was high because most humans have been previously infected by influenza, we did detect a 2-fold rise in about 50% and 33% of the subjects after ileum or jejunum vaccine release, respectively. By gating on CD27^(high)^, a marker of recently activated cells, we found a population of α4 + β7^(negative)^/α4^+^β1^+^B cells, which have been identified for their ability to home to the nose and bronchi^19^. Of those, approximately 30% were surface IgA positive and might have contributed to the SIgA response in the nasal samples in this study. These data suggest that a flow cytometric approach has a higher degree of sensitivity for measuring possible vaccine specific mucosal immune responses in humans.

The rAd oral vaccine also generated α4^(high)^β7^(high)^CD27^(intermediate)^ B cells, evidence of a memory mucosal homing response. Influenza vaccine efficacy has traditionally been measured by serum neutralizing antibodies, and the potential contributions of a mucosal response to protection rarely documented, such as in the study by Ambrose, *et al*.^27^. The use of human challenge studies has allowed the importance of IgA responses to be more thoroughly evaluated for protection against disease in several disease models. Following challenge with RSV, Habibi, *et al*., found that the preexisting IgA responses correlated with protection better than serum neutralizing responses, and that RSV infection seemed to be defective in the generation of a memory RSV specific IgA response^28^. Further, in the norovirus challenge studies by Lindesmith, *et al.,* a rapid mucosal IgA response against norovirus was found to correlate with protection against infection^29^. The rapid response is likely to have been induced through stimulation of memory IgA cells, given the speed of the antigen specific response within only a few days after exposure to norovirus. Additional studies in mice have pointed to the importance of the α4^(high)^β7^(high)^CD27^(intermediate)^ phenotype as well; among memory B cells, only α4β7 (but not α4^+^β7^(negative)^) cells gave protection against rotavirus infection in a mouse model^30^. As development of our rAd oral vaccine platform proceeds into a commercial tablet format, the induction of robust mucosal immune responses may be exploited for protection from pathogens that infect and proliferate in the mucosa.

## Additional Information

**How to cite this article**: Kim, L. *et al*. Systemic and mucosal immune responses following oral adenoviral delivery of influenza vaccine to the human intestine by radio controlled capsule. *Sci. Rep.* **6**, 37295; doi: 10.1038/srep37295 (2016).

**Publisher’s note:** Springer Nature remains neutral with regard to jurisdictional claims in published maps and institutional affiliations.

## Supplementary Material

Supplemental Figure

## Figures and Tables

**Table 1:** Geometric Mean Titers and GMFR of HA-specific MN antibodies.

Group	N	GMT Day 0	GMT Day 28	GMFR
ileum	10	14.6	67.2	5
jejunum	10	12.6	83.4	7
