# Supplementary material for: Systemic and mucosal immune responses following oral adenoviral delivery of influenza vaccine to the human intestine by radio controlled capsule
Source: Sci Rep. 2016 Nov 24;6:37295. doi: 10.1038/srep37295 (PMC5121599; doi:10.1038/srep37295)
Supplement: Supplemental Figure [file srep37295-s1.pdf]

Systemic and mucosal immune responses following oral adenoviral delivery of influenza vaccine to the human intestine by radio controlled capsule

Leesun Kim[1], C. Josefina Martinez[1], Katie A. Hodgson[1], George R. Trager[1], Jennifer R. Brandl[1], Erik P. Sandefer[2], Walter J. Doll[2], Dave Liebowitz[1], Sean N. Tucker[1]*

Supplemental Figure

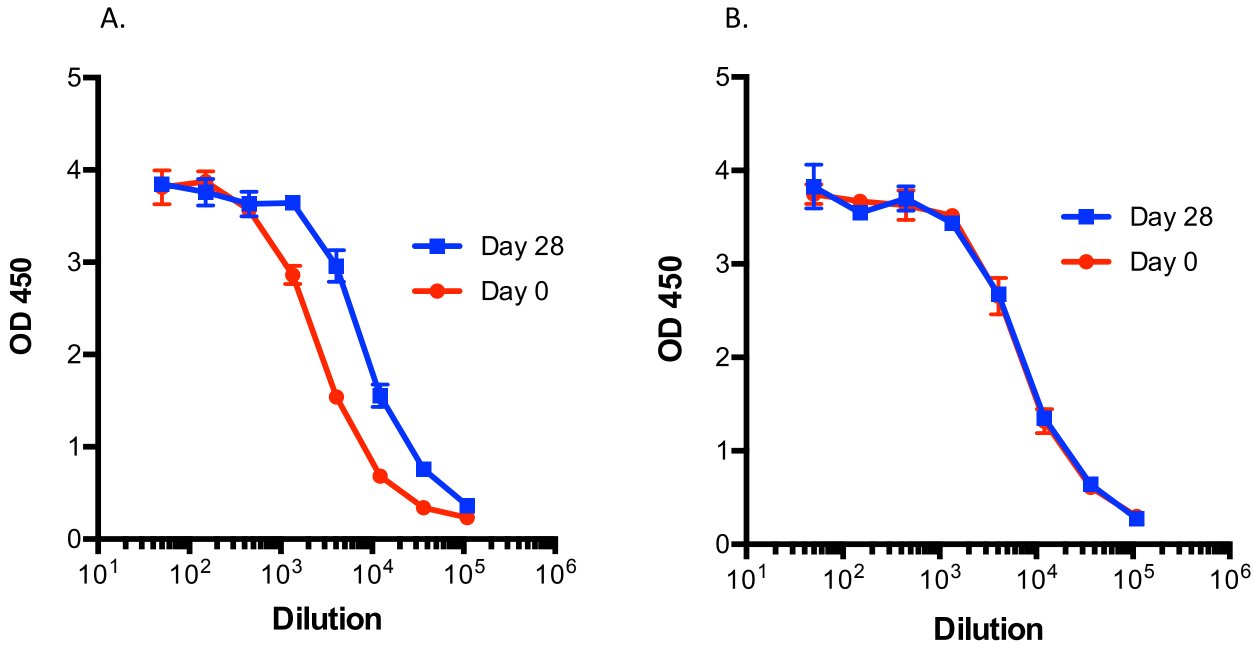

Supplemental Figure: Dilution vs OD curves showing example ELISA responses pre and post immunization for a subject with a detectable increase in the IgG Titers to HA (A) and for a subject with an undetectable response (B).
